# The Influence of Supervisor Developmental Feedback on Employee Innovative Behavior: A Moderated Mediation Model

**DOI:** 10.3389/fpsyg.2019.01581

**Published:** 2019-07-09

**Authors:** Weilin Su, Xinqi Lin, He Ding

**Affiliations:** ^1^School of Labor and Human Resources, Renmin University of China, Beijing, China; ^2^School of Economics and Management, North China Electric Power University, Beijing, China

**Keywords:** supervisor developmental feedback, employee creative self-efficacy, supervisor’s organizational embodiment, employee innovative behavior, moderated mediation model

## Abstract

Previous scholars have recognized the critical role of supervisors in stimulating employee innovative behavior, although it is still unclear whether and how supervisor developmental feedback impacts employee innovative behavior. To resolve this issue, the present study develops and verifies a moderated mediation model to explore the positive influence of supervisor developmental feedback on employee innovative behavior via creative self-efficacy, as well as the moderating role of a supervisor’s organizational embodiment in this process. Analyses of the multi-time data from 375 employees indicate that supervisor developmental feedback is positively associated with employee innovative behavior via his/her creative self-efficacy. Moreover, a supervisor’s organizational embodiment moderates the influence of supervisor developmental feedback on employee creative self-efficacy and the mediating role of creative self-efficacy. From these analyses, the present study not only further develops several views of pervious research in the field of supervisor feedback and employee innovation, but also provides a potential managerial way to promote employee innovative behavior from the perspective of supervisor feedback.

## Introduction

As the competition environment becomes more uncertain, firms that want to survive and develop should rely more on innovative activities (Stroeva et al., 2015; Azar and Ciabuschi, 2017; Zhang et al., 2018). Organizational innovation is becoming increasingly important to gain competitive advantages and realize the sustainable development of firms (Crossan and Apaydin, 2010; Ramirez et al., 2018). As a key factor in improving organizational innovation competence, employee innovation behavior is usually beneficial and has always been regarded as a significant source of competitive advantage for the organization (Shin et al., 2017). Therefore, how to effectively promote employee innovation behavior becomes particularly important at the present stage. Employee innovation behavior is defined as “the new ideas and methods in products and processes generated by employees on the basis of existing conditions,” which includes not only innovative ideas themselves, but also the generation, promotion and realization of innovative ideas (Janssen et al., 2004; Carmeli et al., 2006; Zhang et al., 2018). Many scholars have theoretically and empirically confirmed the effect of the supervisor on employee innovative behavior, which is mainly from the perspective of supervisors’ traits, such as servant leadership (Cai et al., 2018), ethical leadership (Tu and Lu, 2013), transformational and transactional leadership (Pieterse et al., 2010). Nevertheless, such research usually presents the characteristics of fragmentation and functionalization and tends to focus on the active output of supervisors, paying insufficient attention to the interaction between supervisors and employees (Xu et al., 2018).

In fact, supervisor feedback is essential in every organization (Zheng et al., 2015), and is an important form of interaction and communication between supervisors and their subordinates. The employees prefer to seek out feedback from their respective supervisors rather than from colleagues and subordinates (Ashford and Tsui, 1991). Therefore, feedback from supervisors has the most significant impact on employee motivation and behavioral changes compared to the feedback from any other source (Majumdar, 2015), and its influence on employees has been examined by previous researchers (e.g., Steelman et al., 2004; Hon et al., 2013; Zhang et al., 2017). However, traditional supervisor feedback usually only provides evaluative information about employees’ past working behavior and working results (Ilgen et al., 1979), and cannot meet the real needs of employees (Guo et al., 2014). Employees are more welcoming of supportive developmental feedback from their supervisors (Anseel and Lievens, 2007), and it would be wiser for supervisors to affect their employees’ attitudes and behaviors more effectively by providing developmental feedback (Longenecker and Nykodym, 1996). Supervisor feedback is developmental when it provides helpful and useful information that can be used by feedback recipients to improve their current and future work (Zhou, 2003). It can cultivate a creativity-supportive content, in which employees are more likely to proactively engage in innovative activities (Zhou, 2003; George and Zhou, 2007). In addition, the behavioral focused, constructive (as opposed to evaluative or threatening), and learning-oriented developmental feedback from a supervisor can help employees to act in ways that benefit the organization. It requires the supervisors to use formal and informal feedback in the organization to build a supportive feedback environment (Steelman et al., 2004; Dahling et al., 2017). Previous research has indicated that such support from supervisors could encourage employees to pour themselves into innovative activities and exhibit more innovative behaviors (e.g., De Jong and Den Hartog, 2007; Gumusluoğlu and Ilsev, 2009; Wu and Parker, 2017). Hence, in the present study, we attempt to investigate the influence of supervisor developmental feedback on employee innovative behavior from the perspective of supervisor feedback, which is also the key problem we want to solve in the present study.

Supervisor developmental feedback, which refers to the extent to which supervisors provide employees with helpful and useful information that enables employees to learn, develop, and make improvements (Zhou, 2003), has gradually attracted the attention of scholars in the field of innovation. However, there are inconsistent results amongst research on the relationship between supervisor developmental feedback and employee innovation. For example, George and Zhou (2007) found that the direct influence of supervisor developmental feedback on employee creativity was not significant, but Joo et al, (2012) verified that supervisor developmental feedback has a positive influence on team creativity. The reasons that previous scholars come to these inconsistent conclusions are as follows. First, feedback is a dynamic process composed of feedback source, feedback information and feedback receiver. The credibility of the feedback source, the quality and the delivery of feedback information, and the perceptions of feedback receiver work together to produce feedback results (Dahling et al., 2017). Second, for exploring the internal mechanism of supervisor feedback and employee innovation, previous research did not control for other possible interference or substitution factors and ignored other influencing paths (Xu et al., 2018). Third, it assumes that employees have similar perceptions of supervisor feedback, and ignores feedback receiver’s construction and accepting of feedback (Steelman et al., 2004), so that it’s difficult to explain why different employees react differently to the similar developmental feedback from their supervisors. Therefore, existing research cannot reflect the overall effect of supervisor feedback on employee, and the influence of supervisor developmental feedback on employee innovation, especially innovative behavior, needs to be further verified.

In order to fully understand how supervisor developmental feedback affects employee innovative behavior, scholars should not only consider supervisor feedback interventions, but also take individual factors into account. Many prior scholars have acknowledged that employees’ personality characteristics, cognition and attitude would have significant impacts on their innovation behaviors (Feist, 1998; Xerri and Brunetto, 2013). Thus, employee innovation behavior is not only the product of a simple exchange relationship between employees and their respective organizations, but also depends on employee’s cognition and evaluation of his/her own innovative ability (Tierney and Farmer, 2002). Besides, in terms of the impact on an employee, supervisors seem to be in a relatively remote position, so there may be a near-end mediator variable (Schaubroeck et al., 2012) to affect employees’ behaviors. According to the social cognition theory, individual efficiency cognition is an important foundation of his/her actions. Only when employees believe that they can achieve the expected results through their behaviors can they have the motivation to act (Bandura, 1986). In view of the positive impact of creative self-efficacy on individual innovation behavior and its mediating effect in different situations, which has been verified (Carmeli et al., 2006; Hsu Michael et al., 2011; Grosser and Venkataramani, 2017), another aim of the present study is to explore the mediating role of creative self-efficacy between supervisor developmental feedback and employee innovation behavior.

Due to a supervisor’s ability to represent the organization, there is an important implicit assumption that the supervisor can influence their respective employees’ attitudes and behaviors (Dai et al., 2018). As a concept that describes the perceptions of employees as to what extent their supervisors can represent the organization, supervisor’s organizational embodiment (SOE) can inevitably promote or weaken the degree to which the supervisors influence their employees’ attitudes and behaviors (Eisenberger et al., 2010, 2014). When employees think that the supervisor can represent their organization, that is they have a high supervisor’s organizational embodiment, they would be more likely to interpret the supervisors’ behaviors as the intention of organization. In this case, the promoting or inhibiting effect of the supervisors on the employees’ attitudes and behaviors will be more obvious (Stinglhamber et al., 2015). Thus, the final aim of present study is to investigate supervisor’s organizational embodiment as a moderating variable to further influence the boundary conditions of the relationships among supervisor developmental feedback, employee’s creative self-efficacy and innovative behavior.

## Theoretical Background and Hypotheses

### Supervisor Developmental Feedback and Employee Innovative Behavior

Zhou (2003) deems that supervisor developmental feedback refers to the extent which supervisors provide valuable and helpful information to their employees, so that the employees can learn, develop and improve their work in the organization. Previous research has supported the argument that when a supervisor offers his/her employee developmental feedback, the employee is essentially engaging in an informational organization practice in nature, and this might lead to the improvement of employee’s attitude, behavior or performance in the future (Zhou, 2003; George and Zhou, 2007; Li et al., 2011; Guo et al., 2014; Joo et al., 2015; Zheng et al., 2015). In the field of innovation, Zhou (2003) verified that the interaction between the employee creative personality and supervisor developmental feedback had a positive impact on employee creativity. George and Zhou (2007) found that positive emotions, negative emotions and supervisor developmental feedback had an interactive effect on employee creativity. When all three levels are high, the employee creativity is the strongest. Joo et al, (2012) confirmed that the interaction effect between developmental feedback and team cohesion was positively associated with team creativity. Hence, similar to prior studies, we infer that supervisor developmental feedback can effectively stimulate employee innovative behavior in the present study, for the following three reasons:

First, supervisor developmental feedback is essentially informational feedback, which can provide useful information for employees instead of making specific job responsibility to improve their performance (Zhou, 2003; Guo et al., 2014). Unlike traditional performance feedback, which focuses on the completion and improvement of the previous task (Joo et al., 2015), supervisor developmental feedback can stimulate the employees interest in the work itself (Joo and Park, 2010). Developmental feedback can enable employees to work in a relaxed and free atmosphere, which could inspire employees’ divergent thinking (Steele et al., 2018). Second, supervisor developmental feedback focuses on learning, developing and improvement, which enables employees to form behavioral guidance with these characteristics (George and Zhou, 2007). In addition, the employees driven by these behavioral orientations, tend to actively seek out challenges. They are more likely to be persistent and unafraid of trial and error (Dweck, 1986). As a result, they are more likely to learn, master and utilize innovative skills and strategies, and actively generate creative ideas to solve problems. Third, supervisor developmental feedback is future-oriented (Li et al., 2011) and can convey a kind of support and encouragement from the organization for employees’ future development, which can also reduce employees’ concerns about the risks associated with innovation, so, they will have confidence in innovation and show more innovative behavior. Considering the above argument, we assume that development feedback from the supervisors can promote employee innovative behavior and offer the following assumption:

*Hypothesis 1: Supervisor developmental feedback will positively influence employee innovative behavior*.

### The Mediating Role of Creative Self-Efficacy

Self-efficacy in a specific field can predict the behavior and performance in this field more effectively (Malik et al., 2015). Creative self-efficacy is a specific form of self-efficacy, which refers to an individual’s belief in his/her ability to creatively complete tasks and achieve creative results (Tierney and Farmer, 2002). It has a significant positive effect on employee innovation behavior and predicts innovation behavior better than any other kind of self-efficacy (Hsu Michael et al., 2011; Newman et al., 2018). Meanwhile, creative self-efficacy is not invariable and can be guided and promoted by external factors (Bandura, 2006; Gong et al., 2009). For example, Mittal and Dhar (2015) confirmed that transformational leadership can promote employee creative self-efficacy and then enhance his/her creativity. Malik et al. (2015) indicated that extrinsic rewards of the organization could be effective in generating employee creative performance via creative self-efficacy.

Previous research has suggested that supportive feedback and supervisor support can promote individual creative self-efficacy (Tierney and Farmer, 2004) and, in line with this argument, we can infer that supervisor developmental feedback can enhance employee creative self-efficacy. As a form of positive feedback that focuses on learning, development and improvement (Zhou, 2003; Zheng et al., 2015), supervisor developmental feedback neither emphasizes the evaluation of employees, nor puts forward specific requirements on their work results (Shalley and Gilson, 2004; Joo et al., 2015), so that it can bring positive emotional experience to employees, make them more confident in their innovation ability, and then elevate their creative self-efficacy. Meanwhile, supervisor developmental feedback emphasizes the initiative to provide employees with information to help them further learn and improve (Li et al., 2011; Guo et al., 2014), which is conducive to employees’ acquisitions of knowledge and skills, as well as the improvement of their own abilities (Gong et al., 2013). Since creativity cannot be stimulated without skills and abilities in relevant fields, job-related knowledge is regarded as an important antecedent variable of creative self-efficacy (Jaussi and Randel, 2014). Therefore, employees receiving developmental feedback from supervisors are more likely to feel confident about their innovation abilities and show higher creative self-efficacy.

Furthermore, there has been a general consensus on the positive relationship between creative self-efficacy and employee innovative behavior (Beghetto, 2006; Hsu Michael et al., 2011; Newman et al., 2018). When difficulties and obstacles arise in the process of innovation, employees with low creative self-efficacy usually adopt emotion-focused processing strategies to generate the motivation to escape from this situation and ultimately form the behavioral orientation of avoiding risks and maintaining the status quo. Conversely, those employees with high self-efficacy always adopt problem-focused coping strategies, generate motivation to actively respond to problems, and form behavioral guidance to adapt to changes and challenge the status quo. Therefore, we conclude that high creative self-efficacy can stimulate employee innovative behavior orientation. To sum up, the present study believes that supervisor developmental feedback can motivate employees to engage in innovative activities and enhance their innovative behavior by promoting their creative self-efficacy. In other words, we infer the mediating role of creative self-efficacy in the relationship between supervisor developmental feedback and employee innovative behavior, so, we propose:

*Hypothesis 2: Creative self-efficacy will mediate the positive influence of supervisor developmental feedback on employee innovative behavior*.

### The Moderating Role of Supervisor’s Organizational Embodiment

The supervisor’s organizational embodiment is defined as the degree to which employees perceive their leaders or supervisors as an “organizational agent,” that is, the degree to which employees identify their leader or supervisor with the organization (Eisenberger et al., 2010). It is rooted in whether employees can be cared and valued by leaders or supervisors and interpreted as the specific basis for how the organization evaluates their contributions, finally determines the degree of exchanging with the organization (Eisenberger et al., 1986). Generally speaking, the supervisors who are interpreted by employees as organizational agents are often perceived to have more disposable resources, thus their organizational status and power will be magnified and their influence on employees will naturally be enhanced (Eisenberger et al., 2014). This means that employees with high supervisor’s organizational embodiment are more likely to interpret the exchange relationship between them and their supervisors as positive, and perceive some certain behaviors of supervisors as organizational behaviors. So, if they receive those positive supervisor behaviors, they are more likely to show positive attitudes, behaviors and so on.

Many previous studies have confirmed that supervisor’s organizational embodiment plays an important moderating role in the process of supervisor behavior style, influencing employees’ psychology, attitude and behavior. Eisenberger et al. (2014) have confirmed that the influence of leader-member exchange on employee affective organizational commitment is more obvious among those employees with high supervisor’s organizational embodiment. They have also verified that if the employee has high supervisor’s organizational embodiment, abusive supervision is positively associated with perceived organizational support (Shoss et al., 2013). Adopting similar logic here, we suggest that the positive influence of supervisor developmental feedback on employee creative self-efficacy will be strengthened by supervisor’s organizational embodiment. Specifically, the employees with high supervisor’s organizational embodiment, are more likely to utilize supervisor developmental feedback to help them learn new knowledge and skills, and actively apply these new skills and knowledge to improve their work, thus enhancing their confidence in innovation. On the contrary, the employees with low supervisor’s organizational embodiment, don’t think supervisors could represent the organization. For them, the supervisors are less attractive and legitimate, and they attach little importance to the developmental feedback from the supervisor and, therefore, supervisor developmental feedback has a weak promotional effect on their creative self-efficacy. In sum, we propose the moderating role of supervisor’s organizational embodiment in the relationship between supervisor developmental feedback and employee creative self-efficacy to be as follows:

*Hypothesis 3: Supervisor’s organizational embodiment will moderate the influence of supervisor developmental feedback on employee creative self-efficacy, such that this influence will be more positive when employee has high level of supervisor’s organizational embodiment and less positive when employee has low level of supervisor’s organizational embodiment*.

**FIGURE 1:**
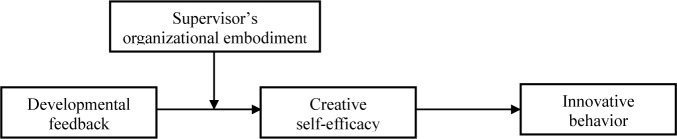
The hypothesized model.

Based on hypothesis 2 and hypothesis 3, we expect that supervisor’s organizational embodiment could also moderate the mediating role of creative self-efficacy in the relationship between supervisor developmental feedback and employee innovative behavior. Specifically, the indirect influence of supervisor developmental feedback on employee innovative behavior via creative self-efficacy should be more significant for employees with a higher level of supervisor’s organizational embodiment than those with a lower level, which is called moderated mediation (Muller et al., 2005; Hayes, 2015). Taken together, we have built a moderated mediation model for the influence of supervisor developmental feedback on employee innovative behavior, as shown in Figure 1.

## Materials and Methods

### Participants and Procedure

In the present study, our data were collected from four companies in Beijing, China, by means of convenience sampling. All the participants are Chinese. With the help of company’s human resource managers, we got a list of all staff names and their email addresses. We randomly selected 400 volunteers and sent them the email questionnaires, with a shopping coupon as an incentive. We also asked the participants to return their questionnaires after completion within a week. Besides, all surveys are anonymous and we promise participants that all their information will be kept confidential.

Since common method bias may inflate the correlations among variables and reduce the accuracy of our conclusion, we collected our data at two different times. In the first wave, we invited participants to fill out questionnaires with dependent variable (innovative behavior), independent variable (supervisor developmental feedback) and demographic questions. One month later, we sent questionnaires containing moderating variable (supervisor’s organizational embodiment) and mediating variable (creative self-efficacy) in the second wave. After removing those samples with incomplete information and fuzzy information, we ultimately retained 375 valid samples (with response rate 93.75%). Besides, in order to further increase confidence in our final valid samples and test whether our results are representative, we used GPower (Faul and Erdfelder, 1992) to conduct *post hoc* power analysis.

Among the valid participants, 41.1% were male, 58.9% were female. For age, 57.3% were under 35 years, and 99.5% were under 45 years. Moreover, most of the participants were well educated, 74.7% of them had at least of a bachelor’s degree or higher. For the average number of years with supervisor, 52.3% of participating employees had worked for less than 4 years, and 79.7% of them had worked for less than 6 years.

### Measures

Based on the aims of this study, we built the theoretical model containing four core variables, which was composed of an independent variable (i.e., supervisor developmental feedback), a dependent variable (i.e., innovative behavior), a mediating variable (i.e., creative self-efficacy) and a moderating variable (i.e., supervisor’s organizational embodiment). All survey items were originally developed in English, so we invited two bilingual scholars (English-Chinese) to translate all items into Chinese (Mandarin) and then back into English following the commonly used back translation procedure.

### Supervisor Developmental Feedback

Supervisor developmental feedback was measured with a 3-item scale developed by Zhou (2003). This scale was used for evaluating the employee’s perception of developmental feedback from his/her direct supervisor. In order to better conform to the language habits of the Chinese employees, we changed the reverse scored item, “My immediate supervisor never gives me developmental feedback,” to “My immediate supervisor often gives me developmental feedback.” All respondents were invited to rate statements from strongly disagree to strongly agree, indicated from 1 to 5, on a 5-point Likert-type scale, according to their actual perceptions of developmental feedback from their direct supervisors. The Cronbach’s alpha for this measure was 0.76.

### Creative Self-Efficacy

Creative self-efficacy was measured with a 4-item scale designed by Tierney and Farmer (2002). A sample item was, “I think I am good at generating new ideas.” All respondents were invited to rate statements from strongly disagree to strongly agree, indicated from 1 to 5, on a five-point Likert scale, based on the extents of their agreement. The Cronbach’s α for this measure was 0.81.

### Supervisor’s Organizational Embodiment

Supervisor’s organizational embodiment was measured with a 9-item scale designed by Eisenberger et al. (2010). The employees evaluated their perceptions of respective supervisors’ sharing characteristics with their organization and the experiences of treatment received from the supervisor as treatment from the organization. A sample item was, “When my supervisor is satisfied with my work, I believe the organization is also satisfied with my work.” All respondents were invited to rate statements from strongly disagree to strongly agree, indicated from 1 to 5, on a five-point Likert scale, based on based on their perceptions of respective supervisors. The Cronbach’s α for this measure was 0.91.

### Innovative Behavior

Innovative behavior was measured with a 9-item scale designed and developed by Janssen (2000, 2003). It contents three sub-scales (innovative ideas generating, promoting and realizing) with three items respective. The samples items were: “I would search out new working methods, techniques, or ideas in daily work” (ideas generating), “I would mobilize my support for innovative ideas in daily work” (ideas promoting), and “I would introduce innovative ideas into work environment in a systematic way if I can (idea realizing).” All respondents were invited to rate statements from never to always, indicated from 1 to 5, on a five-point Likert scale, according their situations. The Cronbach’s α for each sub-scale were 0.83, 0.85, 0.86, and 0.87. The Cronbach’s α for the total innovative behavior scale was 0.90.

### Control Variables

In the present study, we controlled several demographic characteristics including gender, age, education and work tenure with your current supervisor, in correspondence with previous research (Zhou, 2003; Guo et al., 2014; Zheng et al., 2015). Gender was coded as a dummy variable (1 = male, 2 = female). Age, education and work-tenure with current supervisor were all divided into five levels.

### Analytical Strategy

We firstly checked the convergent and discriminant validity of our theoretical model using a confirmatory factor analysis (CFA) by Mplus7.2. According to Hu and Bentler (1999), there are five main indexes to measure the model fit: χ^2^/*df*, TLI, CFI, RMSEA, and SRMR. Specifically, the χ^2^/*df* is less than 2.00, TLI and CFI are more than 0.90, RMSEA and SRMR are less than 0.08, which may be accepted and widely supported (Kline, 2011).

Then, we used the hierarchical regression analysis with SPSS to preliminary test the direct influence of supervisor developmental feedback on employee innovative behavior, the mediation of creative self-efficacy in the influence of supervisor developmental feedback on employee innovative behavior, and the moderation of supervisor’s organizational embodiment in the relationship between supervisor developmental feedback and creative self-efficacy (Aiken et al., 1991; Preacher and Hayes, 2008).

Finally, we used bootstrap methods in virtue of PROCESS program developed by Preacher et al. (2007) with Model 7 to further verify the whole moderated mediation model. We bootstrapped with 5000 in the present study so as to generate bias-corrected confidence intervals of yield 95%. Only the confidence interval excludes 0, and the moderation of supervisor’s organizational embodiment on the effect of supervisor developmental feedback on employee innovative behavior via creative self-efficacy is significant (Hayes, 2015).

## Results

### Confirmatory Factor Analysis

To check whether supervisor developmental feedback, creative self-efficacy, supervisor’s organizational embodiment and innovative behavior could be mutually discriminated, we used Mplus7.2 to conduct the CFA. We compared the four factors model with two three-factors models, a two-factors model and one-factor model, and assessed overall models fitted by goodness-of-fit including, χ^2^/*df*< 3, RMSEA < 0.08, CFI > 0.9, TLI > 0.9, SRMR < 0.08. The results, which is presented in Table 1, show that the four-factors model (Model 1: χ^2^/*df* = 2.73, CFI = 0.91, TLI = 0.90, RMSEA = 0.07, SRMR = 0.07) is better than any other alternative construct models. Meanwhile, the CFA results also indicate that the respondents could distinguish all the constructs clearly.

**Table 1:** The result of Confirmatory factor analysis of the models.

Models	Factors	χ^2^/*df*	RMSEA	CFI	TLI	SRMR
Model 1	Four factors: SDF, CS, SOE, IB	2.73	0.07	0.91	0.90	0.07
Model 2	Three factors (1): SDF + SOE, CS, IB	3.54	0.08	0.86	0.85	0.08
Model 3	Three factors (2): SDF, SOE, CS + IB	4.69	0.09	0.80	0.78	0.13
Model 4	Two factors: SDF+SOE, CS + IB	5.39	0.10	0.76	0.74	0.10
Model 5	One factor: SDF +CS + SOE + IB	10.12	0.15	0.51	0.46	0.15

*N = 375; SDF, represents supervisor developmental feedback; CS, represents creative self-efficacy; SOE, represents supervisor’s organizational embodiment; IB, represents innovative behavior.*

### Descriptive Analysis

Table 2 presents means, standard deviations and correlations among the demographic and four core research variables. An inspection of the correlations shows that supervisor developmental feedback was positively related to employee innovative behavior (*r* = 0.21, *p* < 0.01), creative self-efficacy (*r* = 0.43, *p* < 0.01) and supervisor’s organizational embodiment (*r* = 0.42, *p* < 0.01). Meanwhile, employee creative self-efficacy was positively related to innovative behavior (*r* = 0.24, *p* < 0.01) and supervisor’s organizational embodiment (*r*= 0.44, *p* < 0.01). In addition, employee supervisor’s organizational embodiment was positively associated with innovative behavior (*r* = 0.41, *p* < 0.01). Hence, the results of correlation analysis generally supported our hypotheses of the relationship among these main research variables.

**Table 2:** Descriptive analysis and correlations among variables.

Variables	Mean	SD	1	2	3	4	5	6	7
(1) Gender	1.59	0.24							
(2) Age	2.48	0.65	−0.13^∗∗^						
(3) Education	2.85	0.66	−0.02	0.13^∗^					
(4) Work tenure	2.74	2.21	−0.09	0.38^∗∗^	0.19^∗∗^				
(5) SDF	2.48	0.81	−0.05	−0.04	−0.04	0.08			
(6) Creative self-efficacy	2.51	1.07	0.03	0.02	−0.02	0.14^∗∗^	0.43^∗∗^		
(7) SOE	2.56	0.73	−0.02	−0.02	−0.01	0.09	0.42^∗∗^	0.44^∗∗^	
(8) Innovative behavior	3.14	0.84	0.07	−0.06	0.15^∗∗^	0.05	0.21^∗∗^	0.24^∗∗^	0.41^∗∗^

*N = 375; SDF, represents supervisor developmental feedback; SOE, represents supervisor’s organizational embodiment; ^∗^p < 0.05 and ^∗∗^p < 0.01.*

### Hypotheses Testing

The hierarchical regression results of main study variables are presented in Table 3. Hypothesis 1 predicts a positively direct effect of supervisor developmental feedback on employee innovative behavior. The Model 6 of Table 3 shows that supervisor developmental feedback is significantly related to innovative behavior (Model 6: β = 0.21, *p* < 0.001), thus supporting Hypothesis 1.

**Table 3:** Hierarchical regressions for main study variables.

	Creative self-efficacy			Innovative behavior			
Variables	Model 1	Model 2	Model 3	Model 4	Model 5	Model 6	Model 7
Gender	0.04	0.04	0.06	0.06	0.05	0.07	0.06
Age	−0.03	0.01	0.02	−0.09	−0.09	−0.08	−0.08
Education	−0.05	−0.03	−0.04	0.15^∗∗^	0.16^∗∗^	0.16^∗∗^	0.17^∗∗^
Work tenure	0.16^∗∗^	0.11^∗^	0.09	0.07	0.03	0.04	0.02
SDF		0.41^∗∗∗^	0.26^∗∗∗^			0.21^∗∗∗^	0.14^∗^
CS					0.24^∗∗∗^		0.18^∗∗^
SOE			0.27^∗∗∗^				
SDF × SOE			0.12^∗^				
*R*^2^	0.02	0.19	0.28	0.04	0.09	0.08	0.11
Δ*R*^2^		0.17	0.09		0.05	0.04	0.03
*F*	2.29^∗^	17.44^∗∗∗^	20.30^∗∗∗^	3.41^∗∗∗^	7.35^∗∗∗^	6.29^∗∗∗^	7.23^∗∗∗^

*N = 375; SDF, represents supervisor developmental feedback; CS, represents creative self-efficacy; SOE, represents supervisor’s organizational embodiment; ^∗∗∗^p < 0.001, ^∗∗^p < 0.01, ^∗^p < 0.05.*

In order to check whether creative self-efficacy served as a mediator for the association between supervisor developmental feedback and employee innovative behavior, the present study adopted Preacher and Hayes’ (2008) procedure for justifying a mediation effect. To put this in our research’s perspective, firstly, supervisor developmental feedback should be significantly associated with creative self-efficacy. Secondly, after controlling the direct influence of supervisor developmental feedback on employee innovative behavior, the association between employee creative self-efficacy and innovative behavior should be significant. Finally, the indirect influence of supervisor developmental feedback on employee innovative behavior must be significant as well. As Table 3 shows, after controlling the employees’ demographics, the results of Model 6 showed that supervisor developmental feedback was a significant direct predictor of employee innovative behavior (β = 0.21, *p* < 0.001). When adding creative self-efficacy to the model, it also significantly predicted employee innovative behavior (Model 7: β = 0.18, *p* < 0.01), meanwhile, the effect of supervisor developmental feedback on employee innovative behavior was still significant (Model 7: β = 0.14, *p* < 0.05). Hence, we can conclude that creative self-efficacy partially mediated the influence of supervisor developmental feedback on employee innovative behavior, supporting Hypothesis 2.

The theoretical model of our study predicted that supervisor’s organizational embodiment would not only moderate the effect of supervisor developmental feedback on creative self-efficacy, but also moderate the mediating role of creative self-efficacy in the relationship between supervisor developmental feedback and employee innovative behavior, which should satisfy four conditions (Muller et al., 2005; Hayes, 2015): (1) significant influence of supervisor developmental feedback on employee innovative behavior; (2) significant influence of the interaction between supervisor developmental feedback and supervisor’s organizational embodiment in predicating creative self-efficacy; (3) significant influence of employee creative self-efficacy on his/her innovative behavior; (4) significant difference in conditional indirect influence of supervisor developmental feedback on employee innovative behavior via creative self-efficacy, between high and low levels of supervisor’s organizational embodiment employee.

**FIGURE 2:**
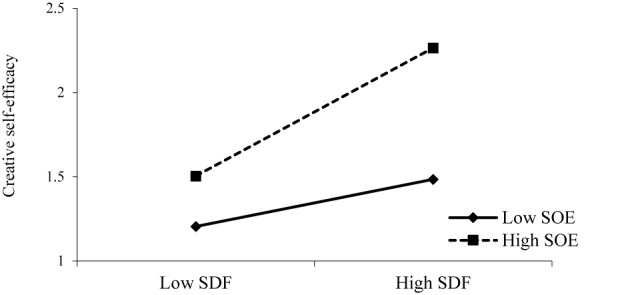
The moderating effect of supervisor’s organizational embodiment on the influence of supervisor developmental feedback on employee creative self-efficacy.

As showed in Table 3, we can test the first three conditions. In Model 6, supervisor developmental feedback was significantly associated with employee innovative behavior, which supported Condition 1. In Model 3, the interaction term for supervisor developmental feedback and supervisor’s organizational embodiment was significant in predicting employee creative self-efficacy, which supported Condition 2. In Model 5, employee creative self-efficacy was positively related to his/her innovative behavior, which supported Condition 3. So, we can conclude that a supervisor’s organizational embodiment could moderate the relationship between supervisor developmental feedback and employee creative self-efficacy, supporting Hypothesis 3. Figure 2 shows this interaction pattern, plotting the relationship between supervisor developmental feedback and employee creative self-efficacy separately for low and high supervisor’s organizational embodiment.

To further test Condition 4, we followed Hayes’ (2013) suggestions by PROCESS macros to examine the whole moderated mediation model. The results indicate that the index value of employee creative self-efficacy for moderated mediation effect is significant [index = 0.02, *SE* = 0.01,95% CI = (0.00–0.05)]. Besides, the employee with a higher level [index = 0.07, *SE* = 0.03, 95% CI = (0.03–0.12)] is more significant than that with a lower level [index = 0.03, *SE* = 0.01, 95% CI = (0.01–0.07)] of supervisor’s organizational embodiment, fulfilling the fourth condition. Therefore, the whole moderated mediation model of our study was fully supported.

Finally, we used the power analysis with GPower to test the final sample size of 375 and 8 predictors as the baseline to further examine whether our valid sample and conclusions were representative and appropriate (Faul and Erdfelder, 1992). Specifically, we based on Cohen’s (1977) suggestions to verify these assessments, which involve in three different effect sizes, small (*f*_2_ = 0.02), medium (*f*_2_ = 0.15), and large (*f*_2_ = 0.35). The results of *post hoc* power analysis revealed that at the 0.05 level the power to detect obtained effect for the whole regression in prediction of employee innovative behavior was 0.86, which was above the value of 0.8 recommended by previous researches (Cohen, 1977; Field, 2009; Mustafa et al., 2016). Hence, we can deduce that our final valid sample of 375 has enough power to detect small effects, and our conclusions based on this sample are appropriate and representative.

## Discussion

Base on supervisor feedback and employee innovation literature, the present study offers theoretical and empirical accounts for whether and how supervisor developmental feedback influence employee innovation behavior by establishing creative self-efficacy as an intervening mechanism and supervisor’s organizational embodiment as a boundary condition. Using multi-time data from a sample of 375 Chinese employees, we found that the influence of supervisor developmental feedback on employee innovative behavior was not only significant, but also mediated by employee creative self-efficacy. In addition, we verified that a supervisor’s organizational embodiment of employee could moderate the relationship between supervisor developmental feedback and employee creative self-efficacy, and the mediating role of creative self-efficacy, such that the more a supervisor’s organizational embodiment of the employees is at work, the stronger this mediating role is. These findings illustrate that supervisor developmental feedback has a positive influence on employee innovative behavior by elevating employee creative self-efficacy, especially when the supervisor’s organizational embodiment is high.

### Theoretical Contributions

The conclusions of present study make several theoretical contributions to the research on supervisor feedback and employee innovation. First, our key problem was to examine the influence of developmental feedback from supervisor on employee innovative behavior. Although research increasingly highlights that a positive supervisor feedback can motivate employees’ positive attitude and behavior by providing useful, helpful and valuable information (Zhou and Shalley, 2008; Belschak and Den Hartog, 2009; Dahling et al., 2017; Zhang et al., 2017), the literature provides scant evidences as to how supervisor developmental feedback affects employee innovative behavior. Our results reveals that supervisor developmental feedback, as a positive and supportive feedback from a supervisor, can promote employee innovative behavior, which is beneficial for the organization. To the best of our knowledge, this is the first study in the supervisor developmental feedback literature that empirically investigates its influences on employee innovation behavior. More specifically, our result demonstrates that the positive influence of supervisor developmental feedback on employee innovation behavior may trigger the employee’s psychological mechanism of creative self-efficacy, which is achieved to a definite extent in our research (Bandura, 2006; Gong et al., 2009; Jaussi and Randel, 2014). This provides the insight that the creative self-efficacy of employee also plays an important role in the process of supervisor feedback and employee innovative behavior. Taken together, for the feedback literature, our research not only heeds the call for the examination of supervisor developmental feedback in a Chinese context (Li et al., 2011; Guo et al., 2014), but also deepens our knowledge and understanding of the influence process of supervisor feedback on employee behavior.

Second, the present study further contributes to the current literature by identifying creative self-efficacy of employee as a mediating mechanism between supervisor developmental feedback and his/her innovative behavior. Previous research has suggested that there were a number of alternative mechanisms in the relationships between supervisor characteristics or behaviors and employee innovative behavior, such as intrinsic motivation (Shin and Zhou, 2003; Shalley and Gilson, 2004; Tu and Lu, 2013), goal self-concordance (Zhang et al., 2017), and meaningful work (Cai et al., 2018), and our results show that creative self-efficacy can be an additional mechanism like them. This means that employees’ motivation to innovate may not only be affected by organizational factors, such as supervisor feedback, but also by their creative self-efficacy (Hsu Michael et al., 2011), which provides a new empirical contribution to the external validity of creative self-efficacy. Meanwhile, previous research suggests that individual creative self-efficacy is the closest factor to employee innovation behavior, and transfers the influence of situational factors on innovation behavior (Gong et al., 2009; Mittal and Dhar, 2015; Grosser and Venkataramani, 2017; Newman et al., 2018). Our study, based on social cognition theory, verified that the effectiveness of creative self-efficacy was an appropriate mediator between supervisor developmental feedback and innovative behavior. This would also be productive for further scholars to explore other potential mechanisms linking situational factors with employee innovation outcomes.

Finally, our results also have some contributions to the supervisor’s organizational embodiment literature by introducing it as a moderator of the relationships between supervisor developmental feedback, employee creative self-efficacy and innovative behavior. Specifically, in employees with high levels of supervisor’s organizational embodiment, developmental feedback from the supervisors may generate more benefits to promote their creative self-efficacy and, thereby, innovative behaviors. For employees with low level of supervisor’s organizational embodiment, regardless of whether supervisors offer them developmental feedback, they are unlikely to take part in innovative activities. That is, high supervisor’s organizational embodiment is essential to determine whether supervisor developmental feedback positively associates with employee creative self-efficacy and innovative behavior. As a new concept, the academic research on supervisor’s organizational embodiment is still in its infancy. Our study introduced it into the field of supervisor feedback and employee behavior for the first time and verified its applicability in Chinese context. Meanwhile, these results have responded to the repeated calls by Eisenberger et al. (2010), Shoss et al. (2013), and Stinglhamber et al. (2015) to investigate the role of supervisor’s organizational embodiment in organizational behavior research and management psychology, and also to shed light on an important boundary condition that strengthens the relationship between supervisor feedback and employee feedback reaction.

### Practical Implications

The present study also provided relevant and fruitful guidance for practitioners and organizations. Firstly, we highlighted the significance of supervisor developmental feedback in promoting employee innovative behavior. Notwithstanding, organizations in a Chinese context usually have a more hierarchical structure than in a Western context (Xin and Pearce, 1996; Cai et al., 2018), so supervisor development feedback still has a positive effect on Chinese employees, with a powerful influence on managing subordinates’ creative self-efficacy and innovative behavior. In this line of thinking, mangers should change their ways of feedback. In daily work, the supervisors should focus on the content of feedback and consciously provide employees with the information they need for their development, learning and improvement, so as to help employees continuously improve their work ability. On the flip side, the supervisor should pay attention to the frequency of feedback, give timely responses and support to their employees, especially regarding new ideas, and guide them to make continuous progress and innovation.

Secondly, our results indicated that employee innovative behavior was not only influenced by supervisor feedback, but also influenced by their own creative self-efficacy. The creative self-efficacy of employee is more closely related to innovative behavior than other external factors (Bandura, 1990; Tierney and Farmer, 2004). Therefore, managers should fully focus on the real demands of employees and constantly stimulate their internal innovation motivation and willingness through various means, to truly encourage employees to put new and creative ideas into practice. In addition, managers should take employees’ characteristics into account, especially when recruiting and selecting newcomers for organization. Recruiters should try to introduce employees with high self-efficacy into enterprises, particularly those positions requiring more innovative behaviors.

Finally, considering the moderating role of a supervisor’s organizational embodiment, diverse management practices should be implemented to increase the levels of supervisor’s organizational embodiment. Specifically, the organization should clarify the legitimacy of the leader’s identity, enhance the internal consistency between supervisor and organization, and truly integrate with each other. Meanwhile, supervisors should conscientiously strengthen their own organizational identity to make the employees really treat them as the embodiment of organization. They also should make their own inner values and external behaviors consistent with the organization to further enhance the employees’ approval and support for them.

### Limitations and Future Directions

Although the present study has several limitations, it does provide some directions for future research. The first one is our sample. We still use employee self-reported assessments of four core variables that may fail to assess them objectively. Even though the CMV in our study weren’t serious, the conclusions should be explained cautiously for the potential CMV caused by the data sources of employee self-assessment. Hence, we encourage future scholars to measure variables at different time from different source (i.e., employees and supervisors). Future research also could use longitudinal designs or quasi-experimental to further improve the accuracy of conclusions.

Second, consistent with previous research, creative self-efficacy (Tierney and Farmer, 2002; Gong et al., 2009) and supervisor organizational embodiment (Eisenberger et al., 2010, 2014; Shoss et al., 2013) of employees are still measured using participant’s self-perception in our study. The self-evaluation of creative self-efficacy may be influenced by biases and under-estimation (or over-estimation), and the employees may not be able to rate accurately the level of organizational embodiment of their supervisors. Therefore, we suggest that further scholars develop new evaluation questionnaires from other sources to evaluate creative self-efficacy and supervisor’s organizational embodiment of employee.

Third, the present research examined employee creative self-efficacy as an intermediary mechanism and supervisor’s organizational embodiment as a boundary condition in the relationship between supervisor developmental feedback and employee innovative behavior and tested the moderated mediating effects simultaneously. However, other mechanisms also could explain this managerial phenomenon. Future scholars could go further by incorporating other mediating or moderating variables, such as intrinsic motivation, employee personality, supervisor support and specific organizational practice.

Fourth, owing to the data selected in China, the generalizability and external validity of our results were limited, especially regarding the West. China is a collectivist culture country. Chinese employees are more concerned about social relationships with their supervisors compared to western employees. They may react differently to developmental feedback from their supervisors. Therefore, we advise future scholars to replicate our research under different cultural contexts. Besides, we also hope that future research about supervisor feedback that are rooted in China could take Chinese culture into account.

Finally, we suggest another possible direction to facilitate research in the field of feedback. The present study has just investigated the influence of supervisor developmental feedback, which is a specific form of feedback, on employee innovative behavior. However, feedback is a complex process (Carless, 2006; Pokorny and Pickford, 2010), and feedback behavior, the credibility of the feedback source, the quality and the delivery of feedback work together to produce feedback results (Dahling et al., 2017). Just discussing a single type of feedback seem cannot fully reveal its consequences. Therefore, future studies could be based on a more comprehensive concept, such as feedback environment (Steelman et al., 2004), to explore the influence of supervisor feedback on employee.

## Conclusion

The present study shows that developmental feedback from a supervisor has a positive influence on employee innovative behavior. In particular, our results indicate that supervisor developmental feedback positively and indirectly associated with employee innovative behavior via creative self-efficacy. Moreover, our results suggest a moderated mediated model, in that, the supervisor’s organizational embodiment of employee not only moderates the direct influence of supervisor developmental feedback on employee creative self-efficacy, but also moderates the mediating role of employee creative self-efficacy in the relationship between supervisor developmental feedback and employee innovative behavior.

## Ethics Statement

The present study was carried out in accordance with the recommendations of the ethics committee of the Renmin University of China with written informed consent from all subjects. All the participants were asked to read and approve this ethical consent before taking part in the present study and follow it in the process of research. The protocol was approved by the ethical committee of the Renmin University of China.

## Author Contributions

WS, XL, and HD were responsible for and took part in this study. WS as the first author, mainly designed the basic model, analyzed the data, and wrote the manuscript. XL made some contributions in data collection. HD took part in research design and data analysis.

## Conflict of Interest Statement

The authors declare that the research was conducted in the absence of any commercial or financial relationships that could be construed as a potential conflict of interest.
